# Immunotherapy for Biliary Tract Cancer in the Era of Precision Medicine: Current Knowledge and Future Perspectives

**DOI:** 10.3390/ijms23020820

**Published:** 2022-01-13

**Authors:** Davide Ciardiello, Brigida Anna Maiorano, Paola Parente, Maria Grazia Rodriquenz, Tiziana Pia Latiano, Cinzia Chiarazzo, Valerio Pazienza, Luigi Pio Guerrera, Brunella Amoruso, Nicola Normanno, Giulia Martini, Fortunato Ciardiello, Erika Martinelli, Evaristo Maiello

**Affiliations:** 1 Oncology Unit, Casa Sollievo della Sofferenza Hospital, 71013 San Giovanni Rotondo, Italy; b.maiorano@operapadrepio.it (B.A.M.); grazia.rodriquenz@gmail.com (M.G.R.); latianotiziana@gmail.com (T.P.L.); cinzia.chiarazzo@gmail.com (C.C.); luigipioguerrera@hotmail.it (L.P.G.); brunellamoruso@gmail.com (B.A.); e.maiello@operapdrepio.it (E.M.); 2 Oncology Unit, Department of Precision Medicine, Università degli Studi della Campania “Luigi Vanvitelli”, 80131 Naples, Italy; giulia.martini@unicampania.it (G.M.); fortunato.ciardiello@unicampania.it (F.C.); erika.martinelli@unicampania.it (E.M.); 3 Department of Translational Medicine and Surgery, Catholic University of the Sacred Heart, 000168 Rome, Italy; 4 Pathology Unit, Fondazione IRCCS Ospedale Casa Sollievo della Sofferenza, 71013 San Giovanni Rotondo, Italy; p.parente@operapadrepio.it; 5 Division of Gastroenterology, Casa Sollievo della Sofferenza Hospital, 71013 San Giovanni Rotondo, Italy; pazienza_valerio@yahoo.it; 6 Division of Medical Oncology, Università di Bari, 70124 Bari, Italy; 7 Cellular Biology and Biotherapy, Istituto Nazionale Tumori, “Fondazione G. Pascale”-IRCCS, 80131 Naples, Italy; n.normanno@istitutotumori.na.it

**Keywords:** biliary tract cancer, immunotherapy, precision medicine, target therapy

## Abstract

Biliary tract cancers (BTC) represent a heterogeneous and aggressive group of tumors with dismal prognosis. For a long time, BTC has been considered an orphan disease with very limited therapeutic options. In recent years a better understanding of the complex molecular landscape of biology is rapidly changing the therapeutic armamentarium. However, while 40–50% of patients there are molecular drivers susceptible to target therapy, for the remaining population new therapeutic options represent an unsatisfied clinical need. The role of immunotherapy in the continuum of treatment of patients with BTC is still debated. Despite initial signs of antitumor-activity, single-agent immune checkpoint inhibitors (ICIs) demonstrated limited efficacy in an unselected population. Therefore, identifying the best partner to combine ICIs and predictive biomarkers represents a key challenge to optimize the efficacy of immunotherapy. This review provides a critical analysis of completed trials, with an eye on future perspectives and possible biomarkers of response.

## 1. Introduction

Biliary tract cancers (BTC) represent a heterogeneous and aggressive group of tumors that originate from the epithelium of intrahepatic (IHCC), extrahepatic (EHCC), distal biliary tree, or from the gallbladder (GBC) [1]. The incidence of BTC differs from the various geographic area, being considered rare in Europe and USA, with an incidence of 1–3 cases per 100,000 people, and a major health problem in other countries, including China, Japan, Korea and Thailand with 5.7 to 85 cases per 100,000 people [1,2,3,4].

To date, the only potentially curative treatment is represented by radical surgery. Unfortunately, only 10–15% of tumors at diagnosis are amenable to surgical treatments, with a high rate of recurrence and a five-year survival of 8–30% [5,6]. For a long time, cholangiocarcinoma has been considered an orphan disease with very limited therapeutic options.

In this scenario, a milestone was represented in 2010 by the ABC-02 trial that proved the efficacy of cisplatin plus gemcitabine as the standard of care (SOC) for patients with metastatic BTC [7]. The combinatory chemotherapy, compared with gemcitabine single agent, exhibited a significant advantage in median progression free survival (mPFS) that translates in improved overall survival (OS) (11.7 vs. 8.1 months; hazard ratio [HR], 0.64; 95% confidence interval [CI], 0.52 to 0.80; *p* < 0.001). It took nearly 10 years and numerous negative trials before the BILCAP study and ABC-06 brought new therapeutic possibilities [8,9].

The BILCAP trial provided the first evidence that postoperative capecitabine is associated with improved outcomes than placebo in resected BTC [8]. Recently, the ABC-06 trial established the FOLFOX regimen, based on a small but significant improvement in survival compared with placebo, as the new SOC following failure of cisplatin plus gemcitabine [9]. After these two lines of treatments, no other therapeutic options are currently approved in the unselected population.

In the last decade, a better understanding of the complex cancer molecular biology led to the identification of gene alterations that, by one side, favor cancer cell proliferation and growth, on the other hand, could represent an element of vulnerability. Indeed, the identification of a molecular driver, susceptible to target therapy, allowed the development of a “sartorial” and personalized treatment for a subset of patients with oncogene-addicted tumors, including BTC [10,11].

At the same time, immunotherapy has dramatically changed the therapeutic armamentarium of several malignancies, transforming, in some cases, a disease with a metastatic and poor prognosis into a curable one [12,13]. However, while different gastrointestinal malignancies, including hepatocellular carcinoma (HCC), benefit from immune checkpoint inhibitors (ICIs), the efficacy of immunotherapy in BTC remains limited [1,2,14].

Therefore, the aim of this review is to light on the possible role and place of immunotherapy in the continuum of treatment of BTC with a critical analysis of completed trials, focusing on future perspectives and possible biomarkers of response.

## 2. The Heterogenous Molecular Landscape of Biliary Tract Cancer

**Figure 1:**
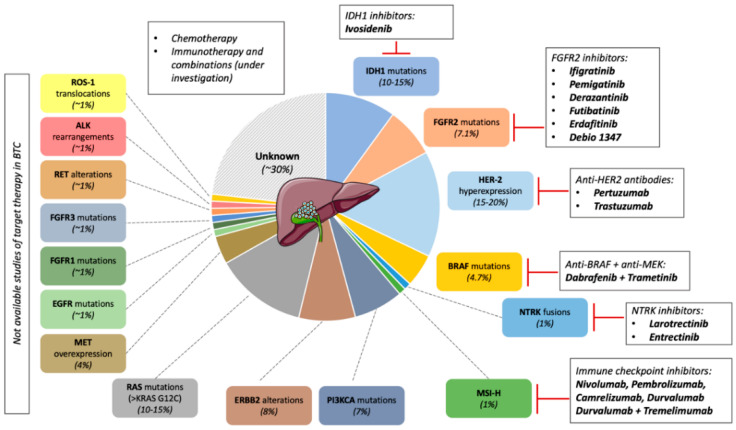
Molecular landscape of biliary tract cancer (BTC). Oncogenic alterations are found in approximately half of the patients diagnosed with biliary tract cancer. So far, only half of the pathways are targetable, with specific inhibitors tested in clinical trials: isocitrate dehydrogenase (IDH)-1, fibroblast growth factor receptor (FGFR)-2, HER-2, BRAF, NTRK. Moreover, patients with microsatellite instable tumors (MSI-H) are prone to respond to immunotherapy. Therefore, for most patients, chemotherapy remains the standard of care; immunotherapy (as single agent or combination) is under investigation.

Due to the limited efficacy of chemotherapy treatments in patients with BTC, major efforts have been necessary to better understand the complex molecular landscape of cholangiocarcinoma (CCA) to identify novel therapeutic targets [15]. Of note, alongside gastrointestinal stromal tumor, melanoma and non-small cell lung cancer, CCA is considered one of the cancers with the highest prevalence of oncogenic driver [2,16]. Robust evidence, derived from large genomic profile analysis suggest that around half of CCA displays a clinically actionable oncogenic alteration [15,17,18] (Figure 1).

For the prescreening of the FIGHT-202 clinical study, an extensive molecular characterization has been conducted in more than 1000 samples of BTC using the next generation sequencing (NGS) Foundation One test [18].

Remarkably, 44.5% of the 1206 patients screened in the trial presented a driver alteration that displayed a matched therapeutic agent either under investigation or approved in other tumor types. Most common alterations encompassed *isocitrate dehydrogenase 1* (*IDH1*) mutations (10.2%), *ERBB2* alterations (8.0%), *fibroblast growth factor receptor 2* (*FGFR2*) mutations or rearrangements (7.1%), *PIK3CA* (7.0%), and *BRAF* mutations (4.7%). Less frequent mutations were reported in *NRAS*, *IDH2*, *EGFR*, *KRASG12C*, *MET*, *FGFR3*, *FGFR1*, *RET*, *JAK2*, *ALK*, and *ROS1*. The frequencies of microsatellite instability (MSI-H) and high tumor mutational burden (TMB) level were observed in approximately 1% of the study population.

A distinctive molecular profile is associated with the different anatomic localization of the neoplasm [19]. Interestingly, *IDH1* mutations and *FGFR2* fusions were almost reported in intrahepatic cholangiocarcinoma, while extrahepatic had a higher frequency of *KRAS*, *CDKN2A*, and *BRCA1* mutations; extrahepatic and gallbladder cancer had higher rates of homologous recombination repair deficiency and HER2 overexpression/amplification. Furthermore, IHCCs and GBCs displayed a statistically significant prevalence of potential positive predictive biomarkers for immune checkpoint inhibition (programmed death-ligand 1 [PD-L1] expression, high microsatellite instability, and high tumor mutational burden) compared with EHCCs.

Based on these findings, there is a growing interest in the clinical development of new molecular target agents (MATs). An extensive analysis of the evolving scenario of target therapies in BTC is beyond the aim of this article and has been elegantly reviewed elsewhere [20,21]. Hereunder are briefly summarized the results of the principal completed studies.

Mutations of the *isocitrate dehydrogenase* 1 and 2 (*IDH2*) are observed in 10–25% of patients with IHCC [18,19]. The activation of these genes determines the excessive production of the metabolite D-2-hydroxyglutarate (2-HG), causing epigenetic dysregulation and aberrant cell signaling [22]. The ClarIDHy is a multicenter, randomized phase III trial, evaluating the activity of the IDH1 inhibitor ivosidenib compared with placebo in patients with *IDH1* mutant CCA that progressed to a previous line of therapy [23,24]. Experimental treatment showed a significant improvement in mPFS [2.7 vs. 1.4 months; HR 0.37; 95% confidence interval (CI), 0.25–0.54; *p* < 0.0001] [23]. Long-term survival analysis showed an increase in mOS, 10.3 months for patients that received ivosidenib compared with 7.5 months in the placebo arm, (HR: 0.79; 95% CI: 0.56–1.12; *p* = 0.09) [24].

When adjusted for crossover (43 out 61 patients received ivosidenib after progression), mOS was 5.1 months in the placebo group, determining a statistically significant improvement in survival (95% CI, 3.8–7.6 months; HR, 0.49 [95% CI: 0.34–0.70]; *p*  <  0.001). Treatment was well tolerated with reduced incidence of severe drug-related adverse events (AEs).

FGFR2 rearrangements/mutations occur in 12–17% of intrahepatic cholangiocarcinoma, resulting in increased cell proliferation, tumor growth and metastatization [25]. Different FGFR2 inhibitors (FGFR2i) (ifigratinib, pemigatinib, derazantinib, futibatinib, erdafitinib, Debio 1347) have been tested in refractory CCA, demonstrating encouraging signals of clinical activity with an overall response rate (ORR) ranging between 20 and 40%, mPFS around 5–7 months [20,21,22,23,26,27,28,29,30,31,32,33,34]. Several phase III trials evaluating FGFR2i compared with standard chemotherapy are currently ongoing [35,36,37].

HER2 overexpression/amplification has been described in up to 15–20% of gallbladder cancer and EHCC [1,19]. Recently, the results of the MyPathway HER2 BTC cohort have been published [38]. The study enrolled 39 patients with advanced HER2 overexpressed or amplified BTC who received trastuzumab plus pertuzumab. Nine out of 39 patients achieved a partial response (PR), with an ORR of 23%. Safety was in line with previous findings in other tumor types.

*BRAFV600E* is a well-known driver mutation in melanoma, colorectal, non-small cell lung cancer (NSCLC) and anaplastic thyroid cancer. Of note, nearly 5% of IHCC display BRAFV600E mutations [1,18,19].

The safety and efficacy of BRAF and MEK inhibition in patients with BTC has been investigated in the ROAR study [39]. Forty-three patients with unresectable, recurrent or metastatic pre-treated CCA received dabrafenib plus trametinib. The confirmed PR was 36%, mPFS 9.2 months and mOS 11.7 months. Further and larger studies are required to confirm the signal of activity.

So far, *NTRK1-3* (neurotropic tyrosine kinase receptor) fusions, leading to overexpression and constitutive activation of the chimeric protein, have been identified in numerous tumor types, including BTC [40]. The first-generation TRK (tropomyosin receptor kinase) inhibitors larotrectinib and entrectinib proved a remarkable efficacy with a really high ORR (up to 75%) and durable response in tumors with *NTRK* gene fusions [41,42,43]. Numerous patients with BTC were included in the key clinical trials leading to Food and Drug Administration (FDA) and European Medical Agency (EMA) approvals of larotrectinib and entrectinib, which should be considered for this rare population.

However, if for almost 40–50% of patients there are molecular drivers susceptible to target therapy, for the remaining population, new therapeutic options represent an unsatisfied clinical need. It is known that one of the mechanisms of resistance of BTC to standard is represented by the presence of a highly desmoplastic and immunosuppressive tumor microenvironment (TME) [44]. Cancer cells can hijack the immune system by favoring the activation of cancer activating fibroblast (CAFs), regulatory T cells (Tregs), influencing the polarization of tumor associated macrophages, causing the production of growth factor, suppressive cytokines such as interleukin 6 (IL-6, tumor necrosis factor alpha (TNF-α) and transforming growth factor beta 1 (TGF-β1), thus determining immune escape and inhibiting the immune response [45]. A better understanding of the heterogenous molecular landscape and immunobiology of BTC is warranted for novel treatment development. Nakamura and colleagues performed a gene expression analysis of 260 BTC identifying four subgroups with distinctives profiles [46]. Interestingly in Cluster 4 were included tumors with poor prognosis characterized by the upregulations of immune checkpoint genes (*CTLA4*, *PDC1*, *IDO1*, *LAG3*). A subsequent analysis by Jusakul and colleagues evaluated more than four hundred CCA defining 4 Cluster according on the liver fluke status [47]. Interestingly, Cluster 3 exhibited an overexpression of immune checkpoint genes (*PD-1*, *PD-L2* and *BTLA*) and pathways related to antigen presentation, T cell stimulation and activation. Thus, there is a strong rationale for the use of ICIs in a subset of patients with BTC.

In this scenario, we will discuss the current role and future perspectives of immunotherapy in non-oncogene addicted cholangiocarcinoma.

## 3. Immunotherapy for Biliary Tract Cancer

### 3.1. Immune Checkpoint Inhibitor Single Agent

During the last decade, immunotherapy represented a “Copernican revolution” for the treatment of several malignancies [48]. In fact, the use of immune checkpoint inhibitors (ICIs) blocking PD1/PD-L1 axis is a recognized therapeutic option for several malignancies including NSCLC, melanoma, head and neck squamous cell carcinoma (HNSCC), renal cell carcinoma, urothelial cancer, Merkel cell carcinoma, gastric cancer, esophageal carcinoma, microsatellite instable (MSI-H) colorectal cancer and HCC [49].

Despite initial enthusiasm, monotherapy with PD1/PD-L1 inhibitors displayed limited efficacy in unselected patients with BTC [50] (Table 1).

In a phase 2 single-arm, multicenter study, the antitumor activity of the anti-PD1 nivolumab was assessed in 54 patients with advanced BTC that progressed to at least one line of treatment [51].

The investigator-assessed ORR was 22% (10 out of 46), while the central independent review was 11% (5 out of 46). All patients that experienced a PR had microsatellite stable (MSS) tumors. In the intention to treat population (ITT), mPFS was 3.68 months (95% CI: 2.30–5.69 months) and mOS was 14.24 months (95% CI: 5.98 months to not reached). Expression of PD-L1 was associated with a significant improvement in PFS, however no correlation between PD-L1 expression and OS was reported. The most common grade 3–4 adverse events (AEs) were hyponatremia (6%) and increased alkaline phosphatase (4%).

Unfortunately, these results were not confirmed in the phase I JapicCTI-153098 that evaluated the antitumor activity of nivolumab in refractory BTC [52]. Among 30 patients with pre-treated BTC, mOS was 5.2 months (90% CI: 4.5–8.7), mPFS was 1.4 months (90% CI: 1.4–1.4), only one patient with an MSI-H tumor exhibited a PR.

In the phase Ib KEYNOTE-028 trial, safety and activity of the anti-PD1 pembrolizumab (dose 200 mg every 3 weeks) were investigated in 24 patients with refractory PD-L1 >1% BTC [53]. Three patients of the evaluable population experienced a partial response (PR) as the best response (3/23; 13%). Of note, in one patient with a durable response (>50 months), MSI-H status was detected, while in the other two cases, microsatellite instability was unknown. In the overall population, mPFS was 1.8 months (95% CI, 1.4–3.1); mOS was 5.7 months (95% CI: 3.1–9.8). KEYNOTE-158 is a phase 2 non-randomized study that assessed the activity of pembrolizumab (10 mg/kg every 2 weeks) in 104 patients with advanced with BTC that had received at least one line of therapy [51]. In the study, PD-L1 expression was not mandatory and was assessed retrospectively. The ORR was 5.8% (6/104), all responders had MSS tumors. Median PFS was 2.0 months (95% CI: 1.9–2.1); mOS was 7.4 months (95% CI: 5.5–9.6). Treatment was well tolerated; most AEs were grade (G)1 or 2. Only few patients experienced serious drug-related AEs (KEYNOTE-158, G3-5: 12.5%; KEYNOTE-028, G3-4:16.7%). Similar findings were reported in a phase 2 study assessing the efficacy of the PD-L1 inhibitor durvalumab alone or in combination with the cytotoxic T-lymphocytes associated protein 4 (CTLA-4) inhibitor tremelimumab in pretreated patients with BTC [54]. Among the 42 patients enrolled, two displayed a PR (2/42; ORR 4.8%), mOS was 8.1 months (95% CI: 5.6–10.1) while mPFS was 2 months.

### 3.2. Dual PD-1/PD-L1 and CTLA-4 Inhibition

The combination of PD-1 and CTLA-4 blockade has improved efficacy compared with single-agent ICIs in different tumor types [55,56,57]. Recently, a subgroup analysis of the CA209-538 phase 2 clinical trial that enrolled patients with rare advanced cancers, including patients with BTC, has been published [58]. Between 2017 and 2019, 39 patients with advanced BTC were enrolled and received nivolumab (3 mg/kg) plus ipilimumab (1 mg/kg) every 3 weeks for 4 cycles, followed by nivolumab every 2 weeks. In the ITT population, 9 out of 39 patients exhibited a PR (ORR 23%), with a disease control rate (DCR) of 44%. Interestingly, in line with previous findings, all responses were durable (median duration of response was non reached). All responders had MSS tumors. The mPFS was 2.9 months (95% CI: 2.2–4.6 months), and mOS was 5.7 months (95% CI: 2.7–11.9 months). Treatment was well tolerated, >G2 AEs were reported in 6/39 patients (15%).

In the phase I study (NCT01938612), the tolerability and activity of durvalumab plus tremelimumab were assessed in 65 patients with heavily pretreated patients with BTC [54].

Treatment was feasible, ≥G3 AEs occurred in 23% of cases and led to discontinuation in 5 patients. One drug-related AE, due to liver injury, was observed. The ORR was 10.8% (7/65); median duration of response (DOR) 8.5 months; mOS 10.1 months (95% CI, 6.2–11.4).

### 3.3. ICIs and Transforming Growth Factor Beta (TGFβ) Blockade

Transforming growth factor-beta (TGFβ) is a pleiotropic cytokine that can exhibit a pro-anti/tumor activity in a context-dependent manner [59]. In established tumors, TGFβ displays an oncogenic activity by creating an immune-suppressive tumor microenvironment and inducing tumor growth, angiogenesis, disease progression, and metastatic spread. Activation of TGFβ signaling is correlated with epithelial-mesenchymal transition (EMT), increased aggressiveness and poor prognosis in CCA [60]. Bintrafusp alfa (M7824) is a first-in-class bifunctional antibody, composed of a monoclonal antibody (MaB) targeting PD-L1 joined with the extracellular domain of two transforming growth factor beta receptor II (TβRII) molecules, which act as a ‘trap’ sequestering TGFβ in the TME.

In a phase I, open-label trial, the tolerability of bintrafusp alfa was assessed in 30 Asian patients with BTC who progressed to first-line chemotherapy [61]. Two patients exhibited a complete response (CR) and four a PR (ORR 20%). Interestingly, 5 out 6 responders displayed a DOR more than 12 months. MPFS was 2.5 months (95% CI: 1.3 to 5.6) and mOS was 12.7 months (95% CI: 6.7 to 15.7). Treatment was feasible, and 11 patients (37%) had ≥G3 AEs. Three patients had G5 events: 1 septic shock and 2 interstitial pneumonitis. Recently, the preliminary results of a phase II study (NCT03833661) evaluating the activity of bintrafusp alfa in 159 patients that progressed to first-line platinum-based chemotherapy is currently ongoing. The ORR was 10.1% (95% CI: 5.9% to 15.8%), final survival results are still awaited [62].

### 3.4. Combination of ICIs and Chemotherapy

There is an increasing amount of evidence that the antitumor activity of chemotherapy is not only related to the cytotoxic activity on tumor cells, but also to the hypothetical elicitation of the host immune response [63]. In fact, certain chemotherapy agents could increase the immunogenicity of cancer cells by determining immunogenic cell death (ICD) [64]. The capability of ICD to boost the activation of the immune response relies on release by dying cells of neoantigens and immunostimulatory molecules such as damage-associated molecular patterns (DAMPs) and cytokines [65]. In a pre-clinical model, treatment of different human cancer cell lines with cyclophosphamide, oxaliplatin or gemcitabine, could increase the expression of human leucocyte antigen class 1 (HLA1) on the surface of tumor cells, favoring the activation of cytotoxic T cell (CTL) [66].

Downregulation of major histocompatibility complex (MHC) class I constitutes a mechanism of immune evasion by cancer cells. Different groups have proved that cisplatin could increase the ability of CTLs to identify tumor cells by the upregulation of MHC class I expression [67]. In a human lung cancer model, exposure to sublethal concentrations of the cisplatin plus vinorelbine increased the levels of MHC class I on the tumor cell membrane, augmenting the sensitivity to perforin/granzyme-mediated CTL killing [68].

So far, the combination of chemotherapy plus ICIs is a standard of care for different malignancies, including NSCLC, HNSCC, gastric cancer, esophageal, urothelial carcinoma and breast cancer [69].

Following this rationale, different trials have investigated the addition of ICI to standard chemotherapy (mainly platinum compound plus gemcitabine) (Table 1).

In the phase I JapicCTI-153098, 30 patients with chemo-naïve recurrent/metastatic BTC received nivolumab (240 mg every 2 weeks) plus cisplatin/gemcitabine as first-line treatment [52]. Half of the patients had IHCC, with a good ECOG performance status (PS) (PS0: 83%; PS1: 17%). Median PFS was 4.2 months (90% CI: 2.8–5.6), mOS 15.4 months (90% CI: 11.8–not reached). No CR was reported, while 11 out of 30 patients exhibited a PR by central revision (ORR 37%). Although the treatment was feasible in this highly selected population, no signals of increased activity by addition of nivolumab to chemotherapy backbone was observed [7].

In another phase II study, 32 patients with unresectable or metastatic BTC received cisplatin/gemcitabine plus nivolumab [70]. Of the 27 patients with evaluable responses, 13 (55.6%) exhibited a PR with a DCR of 92.6%. The mPFS was 6.1 months and mOS was 8.5 months.

Recently, the results of a single-arm phase II study assessing the anti PD-1 camrelizumab plus GEMOX (gemcitabine plus oxaliplatin) in patients with advanced BTC, have been published [71]. Of the 54 patients screened, 37 were eligible and received at least one cycle of the experimental strategy. After a median follow-up of 11.8 months (IQR 7.4–19.1), the 6 months PFS rate was 50%, mPFS 6.1 months (95% CI: 5.1–6.8), mOS 11.8 months (95% CI: 8.3–15.4). A slight increase in mPFS (6.9 vs. 5.4 months) and mOS (13 vs. 11.2 months) was observed in gallbladder cancer (15 patients) vs. CCA (22 patients). A promising ORR of 54% (20/37) was observed, with 13 patients (35%) that obtained stable disease as best response and only 3 (8%) that experienced progressive disease.

In a multicenter phase II study conducted in an Asian population of untreated patients with BTC, the activity of camrelizumab plus oxaliplatin-based chemotherapy was investigated [72]. Of the 92 patients included in the study, 29 received camrelizumab plus FOLFOX4 (5-fluorouracil, levocorin and oxaliplatin), while 63 were treated with camrelizumab plus GEMOX. In the general population, 15 out of 92 patients displayed a radiological response (ORR 16.3%). Of note, in the camrelizumab-FOLFOX4 group, the ORR was marginally inferior to the camrelizumab-GEMOX arm (ORR 10.3% vs. 19%). MPFS was 5.3 months (95% CI: 3.7–5.7) in the intention to treat population (ITT), 5.5 months (95% CI: 3.7–6.0) and 3.8 months (95% CI: 3.5–7.3) in the camrelizumab+FOLFOX4 and carmrelizumab+GEMOX respectively. MOS in the overall population was 12.4 months (95% CI: 8.9–16.1), 12.9 months (95% CI: 8.9–17.9) in the FOLFOX4 arm and 13.6 months (95% CI: 5.7–19.1) in the GEMOX arm. Subgroup analysis according to the tumor site showed that camrelizumab+GEMOX was associated with better outcomes in patients with gallbladder cancer (number 11) than in IHCC (number 49). Both treatments were feasible, with no signals of novel toxicities.

At the ASCO annual meeting 2020, the preliminary results of the NCT03046862 study investigating the efficacy of durvalumab +/− tremelimumab and cisplatin/gemcitabine were presented [73]. 121 patients with chemo-naïve BTC were enrolled in 3 cohorts: in the biomarker cohort (BMC) (number = 30) to receive 1 cycle of cisplatin/gemcitabine, followed by the addition of durvalumab from the subsequent cycle of therapy; in a second cohort (number = 45), patients received triple therapy (3C), including cisplatin/gemcitabine plus durvalumab; finally, in the third cohort (number 46), a quadruple therapy (4C) (durvalumab + tremelimumab + cisplatin + gemcitabine) was administered.

Remarkably, ORR was 50% (95% CI: 32.1–67.9) in the biomarker cohort, 73.4% (95% CI: 60.5–86.3) in the triplet arm and 73.3% (60.4–86.2) in quadruplet arm. MPFS was 13.0 months (95% CI: 10.1–15.9), 11.0 months (95% CI: 7.0–15.0) and 11.9 months (95% CI: 10.1–13.7), and median OS was 15.0 months (95% CI: 10.7–19.3), 18.1 months (95% CI: 11.3–24.9) and 20.7 months (95% CI: 13.8–27.6) in the BMC, 3C and 4C respectively. Based on the comparable outcomes of triplet vs. quadruplet therapy, a phase 3 randomized study (TOPAZ-1; NCT03875235) investigating durvalumab plus cisplatin/gemcitabine vs. SOC is currently ongoing (Table 2). The IMMUNOBIL PRODIGE 57 study assessed the combination of durvalumab/tremelimumab plus paclitaxel in patients with BTC that progressed to cisplatin/gemcitabine treatment [74]. Unfortunately, the study was prematurely discontinued because of an unexpected incidence of anaphylactic AEs.

A phase II trial investigated toripalimab combined with gemcitabine and S-1 in Asian patients with advanced BTC (NCT03796429) [75]. Of the 48 eligible patients for evaluation, 13 obtained a PR (ORR 27%), 29 stable disease and 8 disease progression. mPFS was 7.0 month (95% CI: 5.5–9.1 months and mOS was 16.0 months (95% CI: 12.1 to not reached). The most frequent toxicities were leukopenia (92.0%), anemia (86.0%) and rash (50%).

Considering the small activity of ICI monotherapy, several phase 2 and 3 clinical trials assessing the combination of anti-PD1/anti-PD-L1 plus chemotherapy are currently ongoing and are reassumed in Table 2.

### 3.5. Other Strategies to Improve the Efficacy of ICIs

The development of new vessels is a crucial step for tumor growth, progression and spread [76]. During the last twenty years, blocking tumor angiogenesis represented a fundamental field of research. To date, several anti-angiogenic drugs have been approved for cancer treatment, based on the capabilities of inhibiting tumor growth [76,77]. Recently, it has been highlighted the immune-modulatory role of anti-angiogenic therapies [76,78]. In fact, pro-angiogenic cytokines such as vascular endothelial growth factor alpha (VEGF-A), growth factor (PIGF), and hepatocyte growth factor (HGF) are able to induce the proliferation of immunosuppressive cells like myeloid-derived suppressor cells (MDSCs), limiting recruitment and activation of T lymphocytes, and promoting T-cells exhaustion [78]. Based on this strong rationale, combinatory strategies of ICIs with anti-angiogenic drugs have been developed and are now approved for the treatment of HCC and renal cell carcinoma [79,80,81,82].

The combination of the anti VEGF receptor 2 (VEGFR2) ramucirumab with pembrolizumab have been tested in a multicohort phase I study, including patients with pre-treated BTC [83,84]. Unfortunately, the combination therapy showed limited efficacy with an ORR of 4%. mPFS and mOS were 1.6 months and 6.4 months, respectively. More encouraging results were observed with the combination of the anti-PD1 nivolumab or pembrolizumab with the TKI lenvatinib [85]. In a retrospective analysis of a cohort including 56 patients with refractory BTC the ORR was 30.4%, mPFS was 5.0 months (95% CI: 4.0–6.0), and the mOS was 11.0 months (95% CI: 6.6–15.4). A phase II study evaluating the clinical activity of pembrolizumab plus lenvatinib in 100 patients with advanced BTC is currently ongoing (NCT03797326).

Accumulating evidence suggests that radiotherapy could induce ICD and boost immune response activation, representing a good partner in association with ICI [86]. In a pilot study, 15 patients with BTC were treated with durvalumab plus tremelimumab along with radiotherapy to a single metastatic site (three fractions of 8 Gy at cycle 2 every other day). Of note, patients who received radiotherapy exhibited a DCR was 33% with a 17% PR and 8% CR. >G2 AEs were reported in 9 out 15 patients (60%). Considering the limited efficacy of anti-PD1/PD-L1 single agent, a large number of phase I/II trial are currently evaluating novel combinatory strategies including PARP and ATM inhibitors, MEK inhibitors, epigenetic drugs including histone deacetylase modulator and DKK1 antagonist (Table 2).

## 4. Biomarkers

Up to the present time, the use of ICIs demonstrated limited efficacy in an unselected population of BTC, highlighting the necessity of identifying predictive biomarkers of response to improve patients’ selection. Different studies tried to investigate possible markers, established in other tumor types, including microsatellite status, tumor mutational burden and PD-L1 expression [50].

The mismatch-repair (MMR) system has a fundamental role during DNA replication by recognizing and correcting errors in the microsatellite region, thus avoiding and preventing the development of genomic alterations. The presence of a deficit in the mismatch repair machinery (dMMR) leads to an abnormal accumulation of mutations [13]. If, on the one hand, the presence of microsatellite instability favors tumor initiation, on the other hand it represents an element of vulnerability. In fact, the production of excessive amounts of tumor neoantigens could induce the recognition of cancer cells by the immune system. One of the principal mechanisms of immune-escape in MSI-H tumors is the generation of an immune-suppressive TME, characterized by the upregulation of immune on tumor cells. Thus, removing the pedal brake with the use of ICIs could elicit the activation of the immune system [87]. Based on this strong rationale, the use of ICIs has been tested in several tumor types, with deep and durable response [88,89,90,91]. So far, pembrolizumab has received FDA approval for the treatment of patients with advanced solid tumors that have progressed to a prior line of treatment and who have no satisfactory alternative treatment options [92].

In the BTC, the presence of microsatellites instability is infrequent, representing less than 1% of the entire population [18]. Limited data are available in this infrequent subgroup of patients [52,53,93]. In the KEYNOTE-158 study, 233 patients with 27 different non-colorectal MSI-H tumors received pembrolizumab at the dose of 200 mg every 3 weeks [89]. Interestingly, 22 patients with CCA were enrolled. ORR was 40.9% (95% CI: 20.7–63.6), with 2 CR and 7 PR. Median PFS was 4.2 months (95% CI: 2.1 to NR) and mOS 24.3 months (95% CI: 6.5 to NR). Despite encouraging results, more than half the percent of the patients included in this highly selected population did not respond to the treatment and rapidly progressed, indicating the necessity of a better understanding of the complex immunobiology of BTC.

The expression of PD-L1 on tumor cells, macrophages and lymphocytes, was the first biomarker correlated with ICIs efficacy in different malignancies [94]. To date, the predictive role of PD-L1 expression as a marker of response to ICIs in BTC is controversial and has to be clarified.

In the KEYNOTE-028 the presence of PD-L1 expression in ≥1% of tumor and associated inflammatory cells or positive staining in the stroma, was required as an inclusion criterion [53]. In the ITT population, limited activity signals were observed, with only 3 PR (ORR13%) (including one patient with a MSI-H tumor), a very short mPFS and mOS of 1.8 and 5.7 months. Similar results were observed in the KEYNOTE 158 phase II trial that included 104 patients with refractory BTC [53]. PD-L1 expression was retrospectively assessed in 95 cases (61 positive tumors and 34 negative tumors). The patients’ outcomes were not significantly influenced by PD-L1 expression. Among the patients with PD-L1 positive BTC, compared with PD-L1 negative tumors, the ORR was 6.6% vs. 2.9%, mOS 7.2 vs. 9.3 months.

In the phase II study by Feng and colleagues, of the 32 patients included, 12 (37%) had a tumor PD-L1 expression >1, in 14 (44%) cases PD-L1 levels were <1, and in 6 (19%) cases PD-L1 was not evaluable. However, PD-L1 expression was not correlated with improved PFS (*p* = 0.125) [70].

Different findings were recently reported by Chen and colleagues in a phase II trial investigating the combination of GEMOX plus camrelizumab [71]. Four out of five patients (80%) with a tumor proportion score (TPS) of at least 1% had a clinical response, compared with 14 out of 26 (55.8%). Improved mPFS and mOS were observed in patients with TPS ≥ 1, respectively 9 vs. 6 months and 17.8 vs. 11.9 months. Several factors could explain the discordant results between concluded studies, including the small number of patients, heterogeneity and lack of standardization in PD-L1 testing, scoring and analysis. Therefore, large prospective randomized trials are required to assess the role of PD-L1 expression as a biomarker of response.

During the last years, tumor mutational burden (TMB) defined as the total number of somatic mutations per coding area of a tumor genome, emerged as a compiling biomarker for immune-oncology treatments [50,95].

FDA approved pembrolizumab in patients with TMB-high (TMB ≥ 10 mutations/megabase) tumors based on a biomarker analysis of the multicohort, phase 2 KEYNOTE-158 study [96,97]. In the population with TMB of at least 10, an ORR of 29% (95% CI: 21,39) was reported. Of note, in the TMB-high population (number = 104), no patients with BTC were included. Thus, the role of TMB as a marker of response is still investigational for BTC. Through biomarker analysis of the phase 2 study assessing the clinical activity of cisplatin/gemcitabine plus nivolumab, no association between TMB and PFS was observed [70]. Similar results were observed by Chen and colleagues [71].

## 5. Conclusions

For more than a decade, biliary tract cancer has been considered a rare disease with a dismal prognosis and very limited therapeutic options. However, in recent years, a deeper characterization of the complex and heterogeneous molecular landscape of BTC is rapidly changing the therapeutic scenario [15]. Based on recently published data, FGFR2 and NTRK fusion, IDH1/2 and BRAFV600E mutations, HER2 amplification are now recognized as therapeutic targets [23,24,25,26,27,28,29,30,31,32,33,34,35,36,37,38,39,40,41,42,43]. Consequently, NGS testing and inclusion in clinical trials, where target therapy is not available, is highly recommended.

Nevertheless, while precision medicine is moving straight forward, progress in immune-oncology were significantly slower [50]. Despite a very small subgroup of tumors with microsatellite instability (less than 1%) that benefit of ICIs single agent, minimal activity was observed in the vast majority of patients. Therefore, novel combinatory strategies and reliable biomarkers of response are urgently required for improving patients’ selection. To date, the best partner to combine ICIs still has to be determined. Several phase II/III studies are investigating the association of platinum-based chemotherapy in association with anti-PD1/PD-L1 (Table 2). Recently, a press release by Astra Zeneca announced that interim analysis of the phase 3 TOPAZ-1 trials (NCT03875235) showed that the addition of durvalumab to first-line treatment, compared with chemotherapy alone, significantly improved OS and will soon be presented [98]. Other possible strategies, including the association of ICIs with tyrosine kinase inhibitors, anti-angiogenic drugs, target therapies are under evaluation. Due to the small number of patients included in concluded early phase trials assessing the efficacy of immune checkpoint inhibitors in BTC treatment, no predictive biomarker has been validated. Therefore, further translational studies conducted on large phase randomized phase II/III studies are required to better under the immune biology of BTC and identify predictive biomarkers. In conclusion, immunotherapy should not be considered a standard of care in all comers, due to the limited efficacy in unselected population. Nevertheless, signals of clinical activity have been observed, and inclusion in biomarker-guided clinical trials assessing novel therapeutic strategies is fundamental to understand the proper place of ICIs in the continuum of BTC.

## Figures and Tables

**Table 1:** Completed clinical trials assessing the use of immune checkpoint inhibitors (ICIs) for the treatment of biliary tract cancer.

Study Name	Agent	Target	Phase	Patients	Setting	Outcomes
Anti PD-1/PD-L1 monotherapy						
NCT02829918	Nivolumab	PD-1	2	54	Second line and subsequent	mPFS 3.68 months mOS 14.2 months ORR 22%
JapicCTI-153098	Nivolumab	PD-1	1	30	Second line and subsequent	mPFS 1.4 months mOS 5.2 months ORR 3%
KEYNOTE-028	Pembrolizumab	PD-1	1b	24	Pretreated (PD-L1 positive tumors)	mPFS 1.8 months mOS 5.7 months ORR 13%
KEYNOTE-158	Pembrolizumab	PD-1	2	104	Second line and subsequent	mPFS 2 months mOS 7.4 months ORR 5.8%
NCT01938612	Durvalumab	PD-L1	1	42	Second line and subsequent	mPFS 2 months mOS 8.1 months ORR 4.8%
Anti PD-1/PD-L1 combination with CTLA4 inhibitors						
CA209-538	Nivolumab Ipilimumab	PD-1 CTLA4	2	39	Second line and subsequent	mPFS 2.9 months mOS 5.7 months ORR 23%
NCT01938612	Durvalumab Tremelimumab	PD-L1 CTLA4	2	65	Second line and subsequent	mOS 10.1 months ORR 10.8%
Dual PD-L1 and TGFβ blockade						
NCT02699514	Bintrafusp alfa	PD-L1 TGFβ-RII	1	30	Second line and subsequent	mPFS 2.5 months mOS 12.5 months ORR20%
NCT03833661	Bintrafusp alfa	PD-L1 TGFβ-RII	2	159	Second line and subsequent	ORR 10.1%
ICIs plus chemotherapy						
JapicCTI-153098	Nivolumab cisplatin/gemcitabine	PD-1	2	30	First line	mPFS 4.2 months mOS 15.4 months ORR 37%
NCT03311789	Nivolumab cisplatin/gemcitabine	PD-1	2	30	First line	mPFS6.1 months mOS 8.5 months ORR 55.6%
NCT03486678	Camrelizumab Gemcitabine/ oxaliplatin	PD-1	2	37	First line	mPFS 6.1 months mOS 11.8 months ORR 54%
NCT03092895	Camrelizumab Gemcitabine/ oxaliplatin or FOLFOX	PD-1	2	92	First line	mPFS 5.3 months mOS 12.4 months ORR 16.3%
NCT03046862	Durvalumab Cisplatin/ gemcitabine (Biomarker cohort)	PD-L1	2	30	First line	mPFS 13 months mOS 15 months ORR 50%
NCT03046862	Durvalumab Cisplatin/ gemcitabine	PD-L1	2	45	First line	mPFS 11 months mOS 18.1 months ORR 73.3%
NCT03046862	Durvalumab Tremelimumab Cisplatin/ gemcitabine	PD-L1 CTLA-4	2	46	First line	mPFS11.9 months mOS20.7 months ORR73.4%
NCT03796429	Toripalimab Gemcitabine/S-1	PD-1	2	39	First line	mPFS 7 months mOS 16 months ORR27%
Other combinatory strategy						
NCT02443324	Ramucirumab Pembrolizumab	VEGFR2 PD-1	1	26	Second line and subsequent	mPFS 1.6 months mOS 6.4 months ORR 4%
NCT03892577	Pembrolizumab or Nivolumab Levantinib	PD-1 Multi-Tyrosine-Kinase	1	56	Second line and subsequent	mPFS 5 months mOS 11 months ORR 30%
NCT03482102	Durvalumab Tremelimumab Radiotherapy	PD-L1	1	15	Second line and subsequent	ORR 25%

PD-1: programmed death 1; PD-L1: programmed death ligand 1; CTLA-4: cytotoxic T-lynmphocyte-associated antigen 4; mPFS: median progression free survival; mOS: median overall survival; ORR: overall response rate; TGFβ-RII: transforming growth factor beta receptor 2; VEGFR2. Vascular endothelial growth factor receptor 2.

**Table 2:** On-going clinical trials.

Study Name	Agent	Target	Phase	Setting	Number	Primary Outcomes
ICIs plus chemotherapy						
NCT03260712	cisplatin/gemcitabine + pembrolizumab	PD-1	II	1 line	50	PFS rate at 6 months
NCT04003636	cisplatin/gemcitabine + pembrolizumab vs. cisplatin/gemcitabine	PD-1	III	1 line	788	OS
NCT03875235	cisplatin/gemcitabine + durvalumab vs. cisplatin/gemcitabine	PD-L1	III	1 line	757	OS
NCT03478488	KN035 plus gemcitabine/ oxaliplatin vs. gemcitabine/oxaliplatin	PD-L1	III	1 line	480	OS
NCT04172402	Nivolumab + S-1 + gemcitabine	PD-1	II	1 line	48	ORR
NCT04027764	Toripalimab + S-1 + nab-paclitaxel	PD-1	II	1 line	30	ORR
NCT03796429	Toripalimab + S-1 + gemcitabine	PD-1	II	1 line	40	PFS OS
NCT04191343	Toripalimab + gemcitabine + oxaliplatin	PD-1	II	1 line	20	ORR
NCT03785873	Naliri + nivolumab	PD-1	Ib/II	2 line or later	34	Tolerability PFS
ICIs plus tyrosine kinase inhibitor						
NCT03797326	Pembrolizumab + lenvatinib	PD-1 TKI	II multicohort	Pretreated solid tumors including BTC	590	ORR
NCT04211168	Toripalimab + lenvatinib	PD-1 TKI	II	2 line or later	44	ORR Rate AEs
NCT04010071	Toripalimab + axitinib	PD-1 TKI	II	2 line or later	60	PFS ORR
NCT03475953	Regorafenib + avelumab	PD-L1 TKI	i/II	Pretreated solid tumors including BTC	482	Recommended phase 2 dose ORR
Other combinatory strategies						
NCT04057365	Nivolumab + DKN-01	PD-1 DKK1	II	2 line or later	30	ORR
NCT03201458	Atezolizumab + cobimetinib	PD-L1 MEKi	II	2 line or later	76	PFS
NCT03250273	Nivolumab + etinostat	PD-1 HDAC	II	2 line or later	44	ORR
NCT04298021	AZD6738 + durvalumab	PD-1 ATM/ATR	II	2 line or later	74	DCR
NCT03639935	Nivolumab + rucaparib maintenance after platinum-based chemotherapy	PD-1 PARP	II	1 line	35	PFS rate 4 months
NCT03991832	Durvalumab + olaparib	PD-L1 PARP	II	IDH1 mutated tumors including patients with pretreated BTC	78	ORR Overall disease control rate

ICIs: immune checkpoint inhibitors. OS: overall survival; PFS: progression free survival; ORR: overall response rate; DCR: disease control rate. PD-1: programmed death 1; PD-L1: programmed death ligand 1; BTC: biliary tract cancer; TKI: tyrosine kinase inhibitor; AEs: adverse events; MEKi: MEK inhibitor; HDAC: histone deacetylase. ATM: ataxia-telangiectasia mutation. ATR: ataxia telangiectasia and Rad3-related protein. PARP: poly ADP ribose polymerase. IDH1: isocitrate dehydrogenase.

## Data Availability

Not applicable.
